# Artificial intelligence of digital morphology analyzers improves the efficiency of manual leukocyte differentiation of peripheral blood

**DOI:** 10.1186/s12911-023-02153-z

**Published:** 2023-03-29

**Authors:** Ying Xing, Xuekai Liu, Juhua Dai, Xiaoxing Ge, Qingchen Wang, Ziyu Hu, Zhicheng Wu, Xuehui Zeng, Dan Xu, Chenxue Qu

**Affiliations:** 1 Department of Clinical Laboratory, Peking University First Hospital, Beijing, China; 2 Department of Clinical Laboratory, Aerospace Center Hospital, Beijing, China; 3 Department of Clinical Laboratory, Peking University International Hospital, Beijing, China; 4 Department of Clinical Laboratory, Miyun District Hospital, Beijing, China; 5 Department of Clinical Laboratory, Beijing Nuclear Industry Hospital, Beijing, China; 6 Department of Clinical Laboratory, Peking University Shenzhen Hospital, Shenzhen, China; 7 Department of Clinical Laboratory, Shenzhen Traditional Chinese Medicine Hospital, Shenzhen, China; 8 Department of Clinical Laboratory, Huazhong University of Science and Technology Union Shenzhen Hospital, Shenzhen, China

**Keywords:** Accuracy, Artificial intelligence, Digital morphology analyzer, Leukocyte differentiation, Sensitivity, Specificity, Specimen turnaround time, Peripheral blood smear

## Abstract

**Background and objective:**

Morphological identification of peripheral leukocytes is a complex and time-consuming task, having especially high requirements for personnel expertise. This study is to investigate the role of artificial intelligence (AI) in assisting the manual leukocyte differentiation of peripheral blood.

**Methods:**

A total of 102 blood samples that triggered the review rules of hematology analyzers were enrolled. The peripheral blood smears were prepared and analyzed by Mindray MC-100i digital morphology analyzers. Two hundreds leukocytes were located and their cell images were collected. Two senior technologists labeled all cells to form standard answers. Afterward, the digital morphology analyzer unitized AI to pre-classify all cells. Ten junior and intermediate technologists were selected to review the cells with the AI pre-classification, yielding the AI-assisted classifications. Then the cell images were shuffled and re-classified without AI. The accuracy, sensitivity and specificity of the leukocyte differentiation with or without AI assistance were analyzed and compared. The time required for classification by each person was recorded.

**Results:**

For junior technologists, the accuracy of normal and abnormal leukocyte differentiation increased by 4.79% and 15.16% with the assistance of AI. And for intermediate technologists, the accuracy increased by 7.40% and 14.54% for normal and abnormal leukocyte differentiation, respectively. The sensitivity and specificity also significantly increased with the help of AI. In addition, the average time for each individual to classify each blood smear was shortened by 215 s with AI.

**Conclusion:**

AI can assist laboratory technologists in the morphological differentiation of leukocytes. In particular, it can improve the sensitivity of abnormal leukocyte differentiation and lower the risk of missing detection of abnormal WBCs.

**Supplementary Information:**

The online version contains supplementary material available at 10.1186/s12911-023-02153-z.

## Introduction

The morphological examination of leukocytes in peripheral blood is a key part of routine blood examination, and it is also the most difficult and most likely to yield missed diagnosis and misdiagnosis among all blood cell morphological examinations. In 2015, the International Council for Standardization in Hematology (ICSH) issued a recommendation on the standardization of nomenclature and grading of peripheral blood cell morphological features [1]. The Hematology and Body Fluid Group of the Chinese Society of Laboratory Medicine issued its *Guideline for the report standardization of complete blood count test* in 2020 [2]. These guidelines provide a specific basis for morphology professionals to achieve a unified reporting method. However, morphological identification itself is still a complex and time-consuming task, having especially high requirements for personnel expertise. Therefore, blood cell morphology examination has not been carried out in some laboratories, mainly due to the shortage of morphological professionals.

In recent years, the morphological identification of blood cells using artificial intelligence (AI) has gradually made significant progress [3–5]. Different models or algorithms can be used to help with manual leukocyte identification, but their accuracy still needs to be verified [6, 7]. In 2019, ICSH published recommendations for the application of digital imaging technology [8], which summarized the advantages of using AI algorithms to pre-classify cells. More attention needs to be paid to standardization issues and the accuracy of the instrument that does the cell pre-classification.

Aiming to explore the role of AI in the morphological identification of leukocytes, this study used an AI to assist our technologists in the morphological identification of peripheral-blood leukocytes. AI is developed based on a data-driven deep learning model. A large number of cell images were acquired and annotated as the training set, and a convolutional neural network (CNN) model was utilized to gradually mine the shallow and middle features and eventually get the high-dimensional features of the cell images. The extracted high-dimensional features were input into the classifier to obtain the final leukocyte differentiation. Compared to the traditional manual differentiation, the CNN-based method can obtain high-dimensional features from learning a large amount of data and can better describe the cellular information, which should be able to differentiate the cells with better performance. Then the results of identification were confirmed by experienced morphological experts.

This study proved that AI could assist manual leukocyte differentiation, accurate AI pre-classification could shorten the time and improve the accuracy of classification.

## Methods

### Research subjects

The study sample was 102 patients in Peking University First Hospital and Beijing Lu Daopei Hospital between March 2021 and April 2021 were selected, including 52 males and 50 females, with an average age of 47 years. They were enrolled because the results of their routine blood examinations triggered the review rules of the hematology analyzer. Blood smears were prepared for microscopic examination to identify their leukocyte morphology. Among them were 13 cases of anemia, 13 cases of acute leukemia, two cases of myeloproliferative tumors, one case of myelodysplastic syndrome, five cases of mature lymphocytic tumors, six cases of nonhematologic malignancies, five cases of autoimmune diseases, and eight cases of kidney diseases. Furthermore, there were eight cases of abnormal blood cell counts and 41 cases of other diseases. This study was approved by the committee of Peking University First Hospital, the certificate No 2021–088.

### Instruments and methods

#### Apparatus

The morphology of leukocytes was identified with the Mindray MC-100i Cell Morphology Analyzer (image reader). Two hundred leukocytes were collected from each blood smear.

#### Methods

**Fig. 1:**
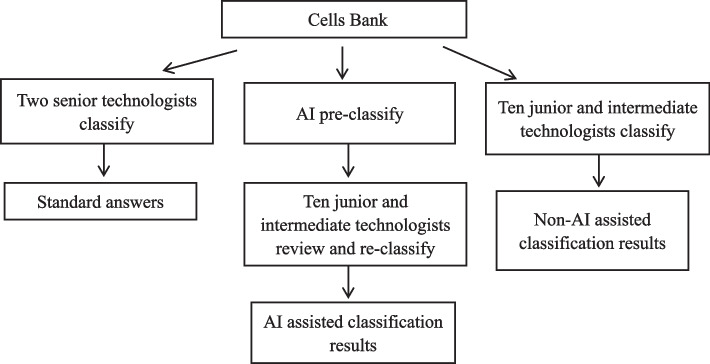
Flow chart of leukocyte differentiation

All blood smear cell images were collected using an MC-100i image reader to form a cell library. All cells were labeled by two morphology experts from a class A tertiary hospital in Beijing with senior professional titles and more than 10 years of experience in morphology, to form the standard answers. Afterward, the image reader used AI to identify and pre-classify all cells. A total of 10 laboratory technologists from different levels of hospitals in Beijing and Shenzhen (level I to level III) were selected, including five with junior professional titles and five with intermediate professional titles. The cell images were first classified by ten technologists on the basis of the AI pre-classification, yielding the AI-assisted classification results. Every technologist performed the test independently. Then, the order of all cells was shuffled to eliminate the identification markers of the AI pre-classification. The same technologists re-identified all cells in the cell bank, yielding the non-AI-assisted classification results (see the Fig. 1). Since the order of all the cells was random, the results of AI-assisted classification have no effect on non-AI-assisted classification. The accuracy, sensitivity and specificity of all normal cells and abnormal cells with and without AI assistance by different laboratory physicians were statistically analyzed (the formulas are as follows). Paired t-test was used for comparison between AI-assisted classification results and non-AI-assisted classification results. Statistically significance was considered when *p* < 0.05.

$$\text{Accuracy}\;({\%})=\frac{\text{Total number of cells correctly identified by physicians among a cell type}}{\text{Total number of cells identified by physicians among a cell type}}\times 100$$

$$\text{Sensitivity}\;\left(\%\right)=\frac{\text{Total number of cells correctly identified by physicians among a cell type}}{\text{Total number of cells marked by experts among a cell type}}\times 100$$

$$\mathrm{Specificity}\;(\%)=\frac{\mathrm{Total}\;\mathrm{number}\;\mathrm{of}\;\mathrm{other}\;\mathrm{cells}\;\;\mathrm{identified}\;\mathrm{by}\;\mathrm{physicians}\;\mathrm{among}\;\mathrm a\;\mathrm{cell}\;\mathrm{type}}{\mathrm{Total}\;\mathrm{number}\;\mathrm{of}\;\mathrm{other}\;\mathrm{cells}\;\mathrm{marked}\;\mathrm{by}\;\mathrm{experts}\;\mathrm{among}\;\mathrm a\;\mathrm{cell}\;\mathrm{type}}\times100$$

## Results

**Fig. 2:**
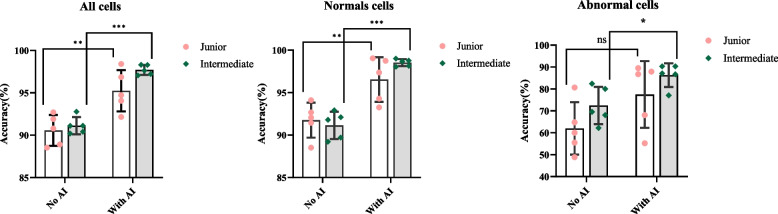
Overall accuracy

**Fig. 3:**
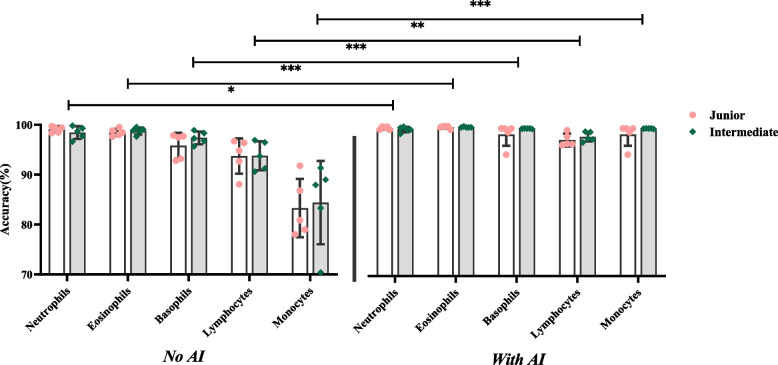
Accuracy of identifying the normal cells

**Fig. 4:**
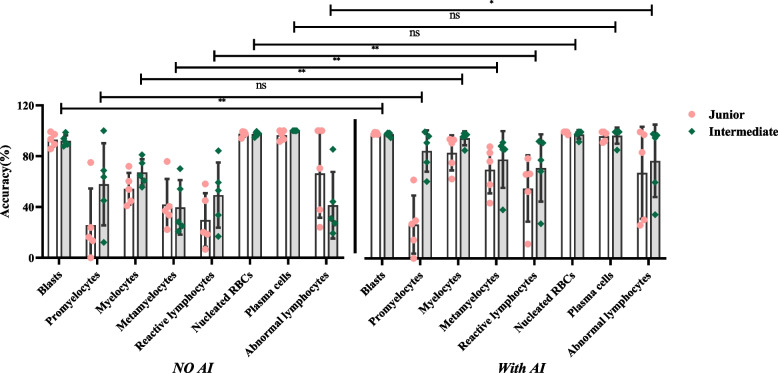
Accuracy of identifying the abnormal cells1

**Fig. 5:**
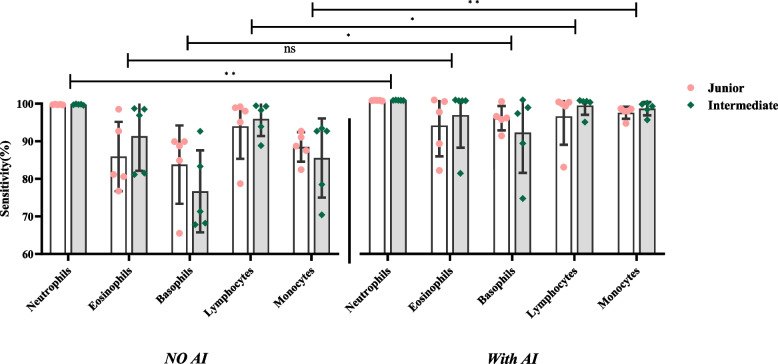
Sensitivity of identifying the normal cells

**Fig. 6:**
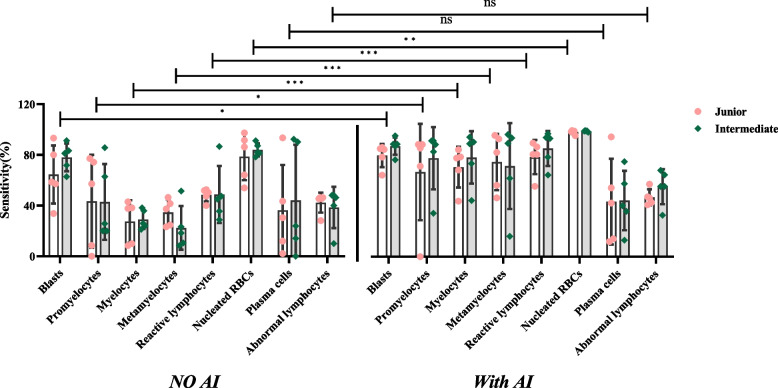
Sensitivity of identifying the abnormal cells

**Fig. 7:**
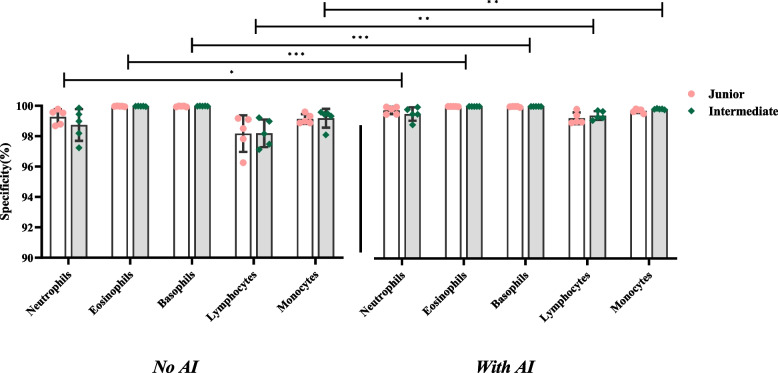
Specificity of identifying the normal cells

**Fig. 8:**
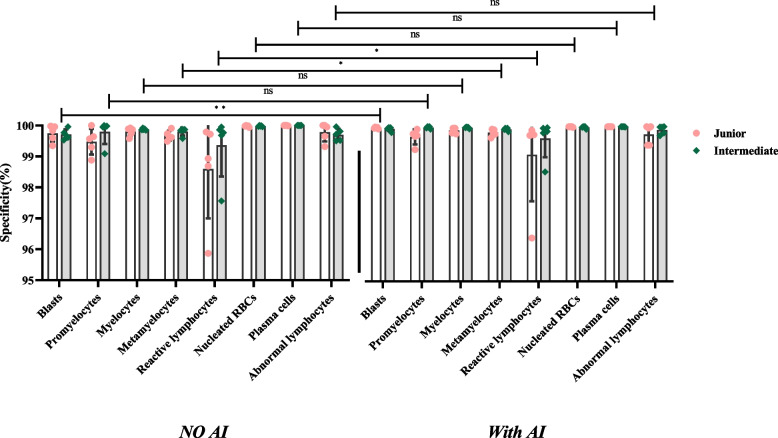
Specificity of identifying the abnormal cells

A total of 23,839 leukocytes were collected, including 21,865 normal cells and 1874 abnormal cells. Normal cells included 5605 neutrophils, 546 eosinophils, 258 basophils, 5208 lymphocytes, and 1106 monocytes. Abnormal cells included 959 blast cells, 36 promyelocytes, 212 myelocytes 142 metamyelocytes, 135 reactive lymphocytes, 226 nucleated red blood cells, 92 plasma cells, and 110 abnormal lymphocytes. The accuracy, sensitivity and specificity of each type of cell are shown in Tables 1, 2, 3, 4, 5, 6 and 7 and Figs. 2, 3, 4, 5, 6, 7 and 8.

**Table 1:** Overall accuracy

Professional title	**All cells**			**Normal cells**			**Abnormal cells**		
	No AI (%)	With AI(%)	Deviation (%)	No AI(%)	With AI (%)	Deviation (%)	No AI(%)	With AI(%)	Deviation (%)
Junior	90.57	95.24	4.67	91.73	96.52	4.79	60.34	75.50	15.16
Intermediate	91.12	97.70	6.58	91.52	98.52	7.40	71.47	86.01	14.54

**Table 2:** Accuracy of identifying the normal cells

Professional title	Neutrophils		Eosinophils		Basophils		Lymphocytes		Monocytes	
	No AI (%)	With AI (%)	No AI (%)	With AI (%)	No AI (%)	With AI (%)	No AI (%)	With AI (%)	No AI (%)	With AI (%)
Junior	99.09	99.68	98.45	99.84	95.66	98.40	93.48	97.20	83.13	94.29
Intermediate	98.43	99.38	98.73	99.85	97.34	99.58	93.67	97.89	83.51	96.61

**Table 3:** Accuracy of identifying the abnormal cells1

Professional title	**Blasts**		**Promyelocytes**		**Myelocytes**		**Metamyelocytes**	
	No AI (%)	With AI (%)	No AI (%)	With AI (%)	No AI (%)	With AI (%)	No AI (%)	With AI (%)
**Junior**	91.38	98.94	10.86	23.08	55.32	84.25	38.99	70.42
**Intermediate**	91.97	98.15	23.66	80	68.14	95.96	40.66	83.28
Professional title	**Reactive lymphocytes**		**Nucleated RBCs**		**Plasma cells**		**Abnormal lymphocytes**	
	No AI (%)	With AI (%)	No AI (%)	With AI (%)	No AI (%)	With AI (%)	No AI (%)	With AI (%)
**Junior**	16.46%	33.21%	97.48%	99.55%	97.66%	98.52%	47.65%	46.49%
**Intermediate**	30.38%	56.18%	97.53%	97.99%	100.00%	99.03%	37.46%	71.53%

**Table 4:** Sensitivity of identifying the normal cells

Professional title	Neutrophils		Eosinophils		Basophils		Lymphocytes		Monocytes	
	No AI (%)	With AI (%)	No AI (%)	With AI (%)	No AI (%)	With AI (%)	No AI (%)	With AI (%)	No AI (%)	With AI (%)
Junior	99.75	99.88	85.93	93.37	83.8	95.27	93.98	95.79	88.48	96.71
Intermediate	99.76	99.92	91.36	96.08	76.67	91.55	95.94	98.58	85.53	97.78

**Table 5:** Sensitivity of identifying the abnormal cells

Professional title	**Blasts**		**Promyelocytes**		**Myelocytes**		**Metamyelocytes**	
	No AI (%)	With AI (%)	No AI (%)	With AI (%)	No AI (%)	With AI (%)	No AI (%)	With AI (%)
**Junior**	64.55	79.83	43.43	66.86	27.45	70.66	34.65	74.79
**Intermediate**	78.06	87.3	42.86	77.71	29.06	78.4	22.39	71.55
Professional title	**Reactive lymphocytes**		**Nucleated RBCs**		**Plasma cells**		**Abnormal lymphocytes**	
	No AI (%)	With AI (%)	No AI (%)	With AI (%)	No AI (%)	With AI (%)	No AI (%)	With AI (%)
**Junior**	48.44	78.67	78.67	98.41	36.3	43.48	42.36	46.91
**Intermediate**	48.74	85.48	83.98	99.03	44.13	44.35	38.55	55.27

**Table 6:** Specificity of identifying the normal cells

Professional title	Neutrophils		Eosinophils		Basophils		Lymphocytes		Monocytes	
	No AI (%)	With AI (%)	No AI (%)	With AI (%)	No AI (%)	With AI (%)	No AI (%)	With AI (%)	No AI (%)	With AI (%)
Junior	99.27%	99.75%	99.97%	100.00%	99.96%	99.98%	98.17%	99.23%	99.13%	99.71%
Intermediate	98.74%	99.51%	99.97%	100.00%	99.98%	100.00%	98.19%	99.41%	99.18%	99.83%

**Table 7:** Specificity of identifying the abnormal cells

Professional title	**Blasts**		**Promyelocytes**		**Myelocytes**		**Metamyelocytes**	
	No AI (%)	With AI (%)	No AI (%)	With AI (%)	No AI (%)	With AI (%)	No AI (%)	With AI (%)
**Junior**	99.74%	99.96%	99.48%	99.67%	99.80%	99.88%	99.68%	99.81%
**Intermediate**	99.71%	99.93%	99.80%	99.97%	99.88%	99.97%	99.80%	99.91%
Professional title	**Reactive lymphocytes**		**Nucleated RBCs**		**Plasma cells**		**Abnormal lymphocytes**	
	No AI (%)	With AI (%)	No AI (%)	With AI (%)	No AI (%)	With AI (%)	No AI (%)	With AI (%)
**Junior**	98.60%	99.10%	99.98%	100.00%	100.00%	100.00%	99.78%	99.75%
**Intermediate**	99.36%	99.62%	99.98%	99.98%	100.00%	100.00%	99.70%	99.90%

### Accuracy

Included the overall accuracy (Table 1 and Fig. 2), the accuracy of normal cells (Table 2 and Fig. 3), and the accuracy of abnormal cells (Table 3 and Fig. 4).

The y-axis represents accuracy, and the x-axis represents the two groups with and without AI assistance. The pink circles represent junior title technicians, and the green squares represent intermediate title technicians. Paired t-test was used for comparison between groups. * represents *P*<0.05, ** represents *P*<0.01, and *** represents *P*<0.0002. In Fig. 2, the difference in the accuracy of the results between the two titles is not significant. Still, when AI assistance is present, there is a significant difference between the results of the two groups of title technicians compared to the results without AI assistance (*P*<0.05).

The y-axis represents accuracy, and the x-axis represents the groups with and without AI assistance under each normal cell type. In Fig. 3, the difference in the accuracy of the results between the two titles is not significant. Still, when AI assistance is present, there is a significant difference between the results of the two groups of title technicians compared to the results without AI assistance (*P*<0.05).

The y-axis represents accuracy, and the x-axis represents the groups with and without AI assistance under each abnormal cell type. In Fig. 4, the difference in the accuracy of the results between the two titles is not significant. Still, when AI assistance was present, there was a significant difference in the recognition accuracy of blasts, myelocytes, metamyelocytes, reactive lymphocytes and abnormal lymphocytes compared with no AI assistance (*P* <0.05).

#### Sensitivity

Included the sensitivity of normal cells (Table 4 and Fig. 5) and the abnormal cells (Tables 5 and Fig. 6).

The y-axis represents sensitivity, and the x-axis represents the groups with and without AI assistance under each normal cell type. In Fig. 5, the difference in the sensitivity of the results between the two titles is not significant. Still, the sensitivity of neutrophils, basophils, lymphocytes and monocytes was significantly different in the presence of AI assistances compared with the absence of AI assistances (*P* <0.05).

The y-axis represents sensitivity, and the x-axis represents the groups with and without AI assistance under each abnormal cell type. In Fig. 6, the difference in the sensitivity of the results between the two titles is not significant. Still, the sensitivity of blasts, promyelocytes, myelocytes metamyelocytes, reactive lymphocytes and nucleated RBCs was significantly different in the presence of AI assistances compared with the absence of AI assistances (*P* <0.05).

#### Specificity

Included the specificity of normal cells (Table 6 and Fig. 7) and the abnormal cells (Table 7 and Fig. 8).

The y-axis represents specificity, and the x-axis represents the groups with and without AI assistance under each normal cell type. In Fig. 7, the difference in the specificity of the results between the two titles is not significant. Still, the specificity of all cell types was significantly different in the presence of AI assistances compared with the absence of AI assistances (*P* <0.05).

The y-axis represents specificity, and the x-axis represents the groups with and without AI assistance under each abnormal cell type. In Fig. 8, the difference in the specificity of the results between the two titles is not significant. Still, the specificity of blasts, metamyelocytes, and reactive lymphocytes was significantly different in the presence of AI assistances compared with the absence of AI assistances (*P* <0.05).

#### Time comparison

Without AI assistance, the average time for each technologist to classify a blood smear was 270 s; after AI pre-classification, the average time for each technologist was 55 s. The AI saved the technologists member more than 3 min per blood smear.

## Discussion

In recent years, AI technologies have been gradually more applied to identify peripheral blood cells in clinical practice [9–11]. They can distinguish and pre-classify different blood cells based on their morphological characteristics [12–14]. Due to the complexity and variability of cell morphology, all current techniques cannot accurately identify all normal and abnormal cells, so the results of pre-classification need to be reviewed by experienced morphology experts [8, 15]. Therefore, the scope of the application of AI is not to completely replace humans but to assist them in more accurately and quickly identifying cells [16].

In this study, the results of cell differentiation by experienced morphological experts were used as the gold standard. To eliminate differences caused by personnel and region of practice, 10 laboratory technologists from different regions and with different years of experience were selected to identify and classify peripheral blood leukocytes with and without AI assistance. Whether for normal cells or abnormal cells, regardless of the area where the subject worked and the professional title, the overall accuracy with AI assistance were higher than those without AI assistance, especially in the case of abnormal cells. These results indicate that AI algorithms can help laboratory physicians more accurately identify cells, and in particular, they can improve the sensitivity of abnormal cells, which is critical to avoid missed diagnosis. In addition, AI assistance can significantly shorten the time for technologists to classify blood smears, help improve their work efficiency, and shorten the specimen turnaround time.

Among the normal leukocytes in peripheral blood, the identification of neutrophils benefitted the least from AI assistance, with 0.59% and 0.95% increases in personnel with junior and intermediate professional titles, respectively. Because their morphological characteristics are typical, they can be accurately identified as long as the technologists has basic morphological knowledge. Even without AI assistance, the accuracy for neutrophil identification can reach more than 98%. Other cells benefited to varying degrees from AI assistance. In particular, both the accuracy and sensitivity of monocytes were increased by more than 10%. Because monocytes are more variable in morphology, they are sometimes easily confused with myelocytes and metamyelocytes, so they better reflected the value of the AI pre-classification.

For the abnormal cells that do not appear in the peripheral blood under normal conditions, the results of different cells with or without AI assistance are quite different. The first is the accuracy. With the AI assistance, the most marked improvement was seen in immature granulocytes, including promyelocytes, myelocytes, and metamyelocytes, followed by abnormal lymphocytes, reactive lymphocytes, and blast cells, while the changes in nucleated red blood cells and plasma cells were the least significant. The reasons for these differences are mainly as follows: First, different cells can be identified with more or less difficulty. The stage of development of the three types of immature granulocytes causes difficulty in distinguishing their morphologies. Due to the continuity of cell differentiation and development, cells between the two stages are often seen. This will result in differences in results due to subjective factors characterizing different personnel. Second, the AI had different abilities to identify different abnormal cells. This study found that AI had insufficient ability to identify abnormal lymphocytes, as it easily confused small blast cells with abnormal lymphocytes or classified abnormal lymphocytes as other cells. Because these cells include very complex types, such as hair cells and lymphoma cells, their morphology varies greatly. In clinical practice, it is difficult to identify accurately for even experienced morphology experts. The third is the difference in the morphological identification ability of different personnel. This study found that the accuracy of abnormal lymphocytes decreased by 1.16% in technologists with junior professional titles with AI assistance. The accuracy of identifying abnormal lymphocytes in two of them was reduced with AI assistance, resulting in a lower overall accuracy. However, the accuracy of personnel with intermediate professional titles was significantly improved (34.07%) when they were assisted by the AI, indicating that these two personnel with junior professional titles had insufficient ability to identify abnormal lymphocytes.

For abnormal cells, the sensitivity is more clinically significant than the accuracy, and increasing the sensitivity can help reduce the missed diagnosis rate of abnormal cells. With AI assistance, the sensitivity of abnormal cells in personnel with junior professional titles and intermediate professional titles both increased by approximately 20%. The most obvious changes were for immature granulocytes and reactive lymphocytes, followed by nucleated red blood cells, blast cells, abnormal lymphocytes, and plasma cells. The clinical significance of blast cells is very important, as their presence in peripheral blood is associated with hematological tumors, so they have attracted much attention in clinical work. This study found that with AI assistance, the sensitivity of personnel with junior professional titles and intermediate professional titles increased by 15.29% and 9.24%, respectively, which could significantly reduce the missed diagnosis. Especially when the proportion of blast cells is low, a small number of cells with abnormal morphology may be ignored in manual differentiation, so this is where AI assistance can be of great value.

With AI assistance, the specificity of normal cells and abnormal cells with different professional titles both increased. Since the specificity of each cell without AI is high, the deviation is not particularly significant.

Morphological identification has a certain degree of subjectivity and requires highly competent technologists, which comes with the accumulation of experience. Therefore, this study compared the data of AI-assisted personnel in different regions and with different professional titles (representing accumulated experience). In most cases, with or without AI assistance, the accuracy, sensitivity and specificity of the personnel with intermediate professional titles were higher than those of the junior professionals. Only in very rare cases, such as for metamyelocytes, the sensitivity of personnel with intermediate professional titles was lower than that of junior professionals. After analyzing the data of each person, we found that there was a certain error in the judgment of this type of cell in two personnel from the same hospital in Shenzhen. There was no significant difference at the geographical or hospital level for the identification of other cells.

The performance of the AI itself influenced the AI-assisted cell classification. An accurate AI classification could assist the morphologists in verifying the cells quickly and correctly. However, an incorrect cell classification may lead to a wrong classification result, especially for technicians without enough training and experience.

Leukocyte differentiation is an important part of microscope examination. Accurate classification of white blood cells plays an important role in screening and therapy monitoring for hematology disease. Accurate AI pre-classification helps to reduce the rate of missed diagnosis.

## Conclusion

AI algorithms can assist morphology technologists in identifying peripheral-blood leukocytes, especially in improving the identification accuracy and sensitivity of abnormal cells, shortening the classification time, improving work efficiency, and reducing the missed diagnosis rate of abnormal cells. Due to the need to improve their ability to identify certain cells, AI cannot completely replace manual differentiation, and their classifications still need to be confirmed by experienced morphological experts when encountering difficult cells.

## Supplementary Information

**Additional file 1.**

## Data Availability

All data generated or analyzed during this study are included in this published article and its supplementary information files (raw data).
